# A History of the Mississippi Valley Dental Society

**Published:** 1895-07

**Authors:** E. G. Betty


### THE DENTAL REGISTER.

VOL. XLIX.] JULY, 1895. [No. 7.

### COMMUNICATIONS.

### A History of the Mississippi Valley Dental Society.

BY DR. E. G BETTY.

MR. PRESIDENT AND FELLOW MEMBERS :—I was requested by the Executive Committee to prepare an historical sketch of this Society to present to you at this, the semi-centennial, though in reality the fifty-first meeting of the same.

This is a task I executed nine years ago, presenting the results of my work as an inaugural address when assuming the duties of presiding officer.

It has always appeared to me that some one capable of the effort should undertake the preparation of such a history and I am only sorry that it had not been done by one of those who took an an active part in its organization. The only available material is the minutes contained in the record book, whose faded lines but too surely tell the lapse of time.

More than half a century of busy years, each one leaving its imprint on progress' page have passed into history since the birth of the Mississippi Valley Association of Dental Surgeons.

What was then not much more than a mixture of the callings of the barber and the jeweler, is now become a distinct and noble profession, having for its aim the conservation and integrity of an important part in the economy of the noblest work of God.

The men who thus banded themselves together to further the usefulness of dentistry knew nothing of anæsthesia, the telegraph, the cylinder press or the telephone, all of which now play a most important part in the daily practice of our profession. There were no colleges, no literature, either textual or periodical, no burring engine, no rubber cloth, no cohesive gold, no mineral teeth. What a contrast between the then and now. And all this within so short a time, yet withal so pregnant with great achievement. It almost takes one's breath away to think of it.

The first page of the record book confronts us with the statement that, "In compliance with a call from the 'Cincinnati Association of Surgeon Dentists' in connection with other members of the profession in the West, a convention of Professional Dentists met in Cincinnati on Tuesday, the 13th of August, 1844, at 11 o'clock, A. M., in the lecture room of the Medical College of Ohio." '

There were present at this meeting the following gentlemen: Joseph Taylor, Maysville, Ky.; J. P. Ullery, Lawrenceburg, Ind.; A. D. Bigelow, Newark, Ohio; James Clark, Lebanon, Ohio; D. P. Hunt, Indianapolis, Ind.; G. D. Teter, Ripley, Ohio; D. B. Wheeler, Xenia, Ohio; Wm. B. Ross, Newport, Ky.; F. E. Suire, Madison, Ind.; J. W. Cook, Cincinnati, Ohio; P. Knowlton, Cincinnati, Ohio; A. Berry, Raymond, Miss.; J. B. Ross, Philadelphia, Pa.; M. Rogers, Cincinnati, Ohio; John Allen, Cincinnati, Ohio; H. Crane, Cincinnati, Ohio; Wm. M. Hunter, Cincinnati, Ohio; W. J. Madeira, Cincinnati, Ohio; Charles Bonsall, Cincinnati, Ohio; James Taylor, Cincinnati, Ohio.

A committee of five was nominated to draft a constitution. Drs. Allen, Cook, Rogers, Bigelow and Hunt being the gentlemen chosen. They presented a constitution (which was adopted,) preceded by the following preamble, which, by the way, has rarely, if ever, had its sentiment improved upon by any society since organized.

"The undersigned, practical dentists of the Mississippi Valley, deem it expedient to form an association for the purpose of mutual improvement in the science and practice of our profession. Desirous of promoting the exercise of that gentlemanly courtesy which should characterize members of liberal professions, in both social and professional intercourse, believing also that by frequent interchange of opinions and observations in practice, by reporting from time to time, cases of interest as they occur in individual practice, we may do much to elevate the character and standing of our profession, and make it worthy the confidence of an enlightened public."

Then follows the constitution, which, I believe, has been reconstructed but never definitely acted upon, so that the Society today is practically working without one, a "consummation devoutly to be wished," as such an instrument is by some deemed hurtful to the practical working of a scientific body.

The first address or essay delivered before the newly organized society was by the honored and never to be forgotten James Taylor, his subject being "Medico Dental Education." The address was on motion adopted by the Society and ordered filed among the archives, but which, I am sorry to say, has long since disappeared, unless, indeed, it has been preserved by subsequent publication in the REGISTER, a point upon which I am uninformed, though at this same meeting Drs. Cook, Rogers and James Taylor were appointed a publishing committee, probably for the purpose of printing this and an address by Dr. John Allen on the "History of Dental Science," also read before that meeting.

Thus we have in these simple records the facts pertaining to the organization of one of the very first societies, and to-day the oldest, formed for the purpose of advancing and improving, professional knowledge among the practitioners of dentistry, the vast influence of which could not then be computed by the most enthusiastic, but which is now evidenced by the fifteen thousand or more practitioners in the United States alone, some forty dental colleges and universities, and quite that many periodicals devoted to its interests, and better than all, an enlightened and growing public opinion.

Before the completion of the organization several communications were made, "relating principally to the *mal-practice* of filling teeth with mineral paste."

This naturally leads to the conclusion that there then existed considerable manipulative ability, and the members imagined, with correctness too, that the use of mineral paste, undoubtedly amalgam, would lead to a reduction of professional dignity and standard, to say nothing of commensurate remuneration for performing a legitimate operation. Another instance of the broad views entertained by these pioneers is shown by their resolution to the effect " that the discoveries and improvements known to the members are and ought to be considered the common property of the profession." In 1845, Dr. Wm. B. Ross delivered an address upon the propriety of patenting new remedies and discoveries. A long and animated discussion followed, during which a resolution was presented to the effect that "Any member of this Society who may patent any instrument or mode of practice may be subject to expulsion from the Society." A resolution was also adopted providing that a committee of three be appointed by the chair to investigate and report whether " Dr. Allen, of our Association, is the author of a dental improvement for which he has obtained a patent."

These instances show conclusively that a liberal spirit prevailed, which, if cultivated, would redound to the benefit of the profession at large and very materially hasten its progress. I know of but one instance in which a discovery of vital importance, in fact a foundation rock, was freely given without let or hindrance or price. I refer to Dr. Barnum's gift of the rubber cloth.

This priceless treasure was eagerly accepted ; but how was it rewarded ? Poor Barnum in the generosity of his heart gave away, what to him would have reaped a vast fortune, only to die in poverty. I well recollect that the Dental Profession of the United States was a long, long time collecting the beggarly sum of $1,000 to give as a charity to his widow or to pay his funeral expenses.

However this may be, the spirit was in the right direction and has been the means of building up that fraternal spirit which is now so characteristic of our colleges, our Society meetings and our literature.

At a very early period of its existence the Society made its influence widely felt throughout the medical profession, many members of which were elected to honorary membership, among them was Prof. Daniel Drake, one of the most prominent practitioners and teachers of medicine the West has ever produced, and it was in Cincinnati that he displayed his powers and influence in the advancement of medical and kindred sciences. Our present famous hospital is indebted to him for its establishment, and to him also the Ohio Medical College owes a great deal of its reputation in the early days of this city. His estimation of the scientific ability of the gentlemen composing the Society at this time, 1846, was so high that he sought information from them to incorporate in a work on medicine he was then preparing.

His letter is as follows:

"To the President of the Mississippi Valley Association of Dental Surgeons: *Sir:* — In the Historical and Practical Treatise on our Diseases, which I am now engaged in preparing for the press, I have appropriated a chapter to maladies of the teeth and am anxious to obtain from gentlemen devoted to their treatment as much information as possible. Permit me then to request of the members of the Association over which you preside, such facts and observations as they may be able to communicate on the following points:

First: What is the nature of that diathesis or constitutional predisposition or disorder, if any, which so often occasions decay in the deciduous teeth of our children?

Second: To what causes, external or pathological, local or constitutional, shall we ascribe the premature decay of the second teeth, in the West?

Is a hereditary scrofulous diathesis a cause of infirm teeth? Is dyspepsia a cause of early decay? Does the acid thrown up by many dyspeptics in paroxysms of that disease act chemically on the teeth? What is the effect of repeated salivations on the teeth and gums? What are the effects of tobacco on the teeth, and are those of chewing and smoking the same?

Third: Has the tartar of the teeth a constitutional origin?

Fourth: Is the decay of the teeth greater in the West than in the Atlantic States, in the same latitudes, and is there any difference in different latitudes in the same meridians?

Fifth: Is there any difference as to soundness of teeth between our native and foreign population?

Sixth: Are the teeth of our colored people less subject to decay than those of white, who labor and live in a simple manner as to diet and drinks?

Replies to these questions, or information not referred to in them, on diseases of the mouth generally, communicated to me within the next few months, will be acknowledged as a favor, while I shall scrupulously give with every new or important observation the name of its author.

I have the honor to be your obedient servant,

DANIEL DRAKE."

This letter was read to the Society and a resolution passed appointing a committee to comply with the request as far as possible, and also thanking Prof. Drake for the interest he manifested in promoting dental science.

It may be mentioned in this connection that these very questions in one form or another, have worried the brains of the profession from that day to this, and it is safe to say, have not been adequately and scientifically answered. The man who can do so may rest assured that his name will descend in perpetual honor in the annals of dentistry, and the people will rise up and call him blest.

The Mississippi Valley Society is also responsible for the founding of the DENTAL REGISTER, a journal that has exercised a wide influence in molding professional thought and is still in the ring, not like the prize fighter, "slightly disabled," but strong and vigorous as of yore, ever battling for the right of science and the wrong of ignorance and guided by the sturdy pen and watchful eye of one who has passed his semi-centennial as an investigator, teacher, author and practitioner, the Gladstone of dentistry, Jonathan Taft.

It was in 1846 that the idea of establishing a journal was conceived and found expression in a resolution by Dr. Edward Taylor to the effect "that a committee of three be appointed by the chair to ascertain the cost of publishing a quarterly journal, devoted to the interests of our profession, and, if they think it advisable, to propose a scheme for said publication, and submit it to the next annual meeting of this Society."

The committee appointed to discharge this important trust consisted of Drs. James Taylor, John Allen and Henry Crane, and right well did they execute the commission, for the result of their labors is known wherever there is a practicing dentist. At the evening session of the same day, Dr. Edward Taylor offered an amendment to this resolution, authorizing the committee to issue during the year a specimen number, not to cost in excess of fifty dollars. This, however, was not done, as the first number of "The Dental Register of the West" did not appear until October, 1847, since which time it has not lapsed a single issue, being therefore the oldest dental journal in continuous existence.

It is then, to Edward Taylor, that the profession owes the Register, and not his brother James, as has generally been thought, though the latter was its first editor, and lent to it the energies of an active and well-stored mind.

So long as our Association exists, it may point with pride to its record of usefulness in achieving the two great feats of having been the progenitor of the oldest dental journal in the world and second oldest dental college, though as to the latter, the Society took no action as a body, though the members lent their influence as individuals and provided the means to purchase the ground and erect the building. During its long life the Association usually met in Cincinnati, though upon one occasion it met in Louisville, and another in St. Louis, where a Union Session was held with the Missouri State Society.

Close examination discloses the fact that the records are not complete; part of 1860 is missing. In 1862 no session was held owing to the civil war; the minutes of 1866, 1868 and 1869 are wholly missing, while half of 1871 is absent. This is very much to be regretted, for it detracts from their value as a continuous history of the progress of our profession during the past half century.

The old Society did not always have plain sailing and bright skies, as the casual observer might imagine, judging from the harmony and good feeling that have so long held sway. In one year, 1852, things were at a low ebb, interest seemed to flagg, and indications pointed towards an adjournment *sine die*, thus allowing the College Association to become the medium of professional progress; upon this question, however, the members were equally divided, the proposition was to disband and concentrate upon the above mentioned body. In order to prevent such a calamity, one of the younger members hustled around and found two new members, viz., Dr. George Watt and Dr. John G. Hamill. The accession of these gentlemen produced a reaction, the votes of these latter turned the tide in favor of continuing the Society. It was well they did so, and it is to be hoped that from this day on we will constitute ourselves a committee of the whole to maintain an interest that will perpetuate this dear old Society, the mother of them all.

Many instances of an interesting nature crop out here and there in the pages of the record, but which it is not the province of this paper to treat, though I trust you will pardon mention of one or two, leaving this as an opening for others to relate their anecdotes of bygone days.

During the first years of the Society, a large number of honorary members were elected, generally from the medical profession. At the first meeting it placed upon this list Chapin A. Harris, the father of dental education, and, as Kingsley said: "Fortunate was it for posterity that Chapin A. Harris and his colleagues were denied admission to the medical colleges. They builded wiser than they knew. Dentistry, independent, has grown with a vigor unparalleled."

Professors John Locke, R. D. Mussey and Professor Shotwell, the first a chemist and philosopher of national reputation; the second demonstrated that the skin is an absorbent, while Professor Shotwell identified himself with medical education in this city. I might mention many others, but time presses.

In conclusion I may express the hope that from this time on we will never neglect the old Society nor allow her to die of inanition.

It is a privilege to be a member and a treat to attend her meetings.

Dr. Betty's paper was listened to with close attention.

The Chair then announced that as the hour of adjournment was close at hand discussion of the subject would be deferred necessarily until the morning, and requested all to be on hand promptly at 9 A. M.

Dr. Callahan announced that if the Association should find it impracticable to conclude their sessions on Thursday the hall was at their service indefinitely.

Drs. Emminger and Welch, committee on membership, reported favorably the name of Dr. W. W. Shryock, of Ft. Wayne, Ind., as a candidate for membership in the Association.

On motion the Secretary was instructed to cast the unanimous ballot of the Association admittiug the gentleman to membership, and the Secretary announced the same.

On motion, adjourned until 9 A. M., Thursday, April 18th.

### SECOND DAY—MORNING SESSION.

Convention met pursuant to adjournment, President Taft in the chair.

The Secretary read the minutes of the session of April 17th, which on motion were duly approved.

PRESIDENT TAFT: Is there any miscellaneous business in the mind of any one? A half hour this morning was to be devoted to the paper read yesterday evening by Dr. Betty, on the History of the Society. Four persons are named to open that discussion, the first two of whom, Drs. Morgan, of Nashville, and Butler, of Cleveland, are not present. Dr. Smith comes next.

DR. H. A. SMITH, of Cincinnati: I think it is hardly possible for me to add anything of especial interest, after the very excellent paper upon the history of this Association from the pen of Dr. Betty. Whatever I may say, however, will be in the nature of reminiscences. My advent in the profession was coincident with my joining this Association. The day after receiving my degree I was inducted into this Association, elected a member, and from that time I have without an exception attended each annual meeting. I have often said that if my connection with Dental Associations was blotted out of my professional life it would be rather barren of pleasurable thoughts.

There is a good deal of hard work connected with dentistry, and that is the one bright spot, my relations with my fellows, and especially with Associations like this—especially my relations with my fellow dentists in this Association. At that time I considered it an especial honor to be made a member of the Dental Association, being a young man just from college; and I have always considered it an honor that I have been a member, and so regard it to-day. While I have been connected with a good many Associations, nearly all, in fact, to which I was eligible, yet I regarded my membership here as especially delightful.

On the floor of this body I have seen all the prominent dentists of this country, and it seems to me those of us who were among the early members will recall that we had representatives from New Orleans, and all up and down the Mississippi river proper, such men as the Knapps, of New Orleans; and coming up in this direction we had representatives from Natches, Nashville, Memphis—in fact all the leading men of that section have been members of this Association. From Pittsburg, we used to have, I think, quite a showing of membership, and Cleveland, as well as Cincinnati, and all the States of the Northwest; for instance, such men as Dean, Cushing and Allen. Some of those men are dead; they were in attendance at almost every annual meeting. And we had them from still further West—Milwaukee. Look over dental literature up to the present day, and you will find the leading spirits have been members of the Mississippi Valley Association. Then, is it not an honor to have been a member of such an Association, or, rather, is it not an honor still to be associated in such a body as this? I ought not to omit Professors Watt and Atkinson; some of the most brilliant efforts of Atkinson have been made right on this floor; with his genial manner, always ready to help out a discussion with a joke, as well as with suggestions as to scientific methods, always a welcome member. With these reminiscences, however, I would not detain you longer, except to say that the Mississsippi Valley Association will continue in its usefulness. It has done a good work, certainly, being the pioneer in Association work, and furnishing a pattern for numerous other Associations to follow. I took up an old book the other day, published forty years ago, and I was surprised at the freshness of thought in that journal, as compared with similar publications of to-day. It seems to me that we have not made very much advance. It had a paper by our worthy President on dental carries, a paper by Dr. James Taylor, an article by Dr. Townsend—by the way he has appeared on the floors of this Association. He delivered at one time an address, or two lectures, on the conduct of dental practice, and there is nothing better extant to-day. You can not find anything better. Refer back to all the lectures that were given before this body, say forty-five years ago, and see the uniform excellence of the literature; you will find that contributions from many of the members of this Association live, and bear even to-day the imprint of the best thought; they were the productions of thinking men. That article by Professor Taft struck me, and showed me that we had not made so great an advance over the thought of that day; you will be astonished at what those men investigated. In the paper read by Dr. Betty did you notice the questions presented by Professor Drake to this body—how fresh they were? We are discussing the same topics to-day. The questions Professor Drake put to this body, in order that he might embody the replies in his work, are phenomenal. That indicates how far-seeing and scientific those men were. We ought to inaugurate a movement in this city to build a monument for Dr. Drake, the founder of the Ohio Medical College; he was a medical man, but how thoroughly versed in dentistry. I felicitate myself on the opportunity of continued membership in this Association. I believe it will continue to be a factor of usefulness for many years. (Applause.)

President Taft at this point resigned the chair temporarly to Dr. Brown, and took the floor.

Dr. J. Taft, of Cincinnati: I find my name on the list of those who are to discuss this subject. I do not now remember that I was consulted about it; but I saw this two or three days ago, and so the matter has not, of course, been out of my mind for that time, or, rather, has been occasionally in my mind, and though I have not looked up the literature on this subject connected with the society there are many things that naturally come to the mind of a man when he has been for a long time, a good while, at least, connected with this Association, and took some part in a feeble way in its work, learning more through it however, all the while, than I was ever able to give to it. I remember when coming into this Association in its early days, how much interested I was. I remember how much gratified I was to be associated with the men who constituted its membership, with the men from all over the Mississippi Valley; men then regarded as the leading men of the profession, were embraced in the membership of this body. It was, to a young man coming into association with these men, a matter of considerable pride, to say the least, a matter of great gratification, in which I have no doubt Dr. Smith and many others shared fully. I remember thinking that if I could ever reach the position that many of these men occupied, how proud I should be of my position. They were representative men, of strong character, men who were able in their day to accomplish great work, and they did accomplish such in the organization, and in maintaining and carrying on the labors of this Association. Just think of it! Those men were brought together, fifty to one hundred from all parts of the country, in the early days of the life of this Society, to conduct a work, without a guide, without any precedent. They were to mark out for themselves their line of action; they were themselves to find a way to accomplish a work, in which they had nothing to guide them; and well did they work, as we see, as we know, who have much knowledge of the history of what was done. Now, what did this Society do? The influence of the work of this Society upon numbers of men was very marked, indeed. There were such men as Richardson, whom you well know as the author of mechanical dentistry, and who was trained in this body; and undoubtedly an important stimulus operating upon him in the production of that work was occasioned by the influence of this Society. Dr. Richardson also became a teacher in the Ohio Medical College.

Professor Watt also received much of his training in this Society, and always during his life he was willing to acknowledge his indebtedness to the Mississippi Valley Association. He was always proud to speak of what it had been for him, and to give credit to those who had been instrumental in leading him on to correct ideas, and correct impressions in regard to a professional life. And other men were trained, as well, in the work. These are some of the things that this Society has done. I cannot begin to give you even a synopsis of all the things that were done, but I have referred to a few only. It was the pioneer, and has been actually the pioneer of other societies in this country. The organization of the American Dental Association, the representative body of the profession of this country—none better, perhaps, in the world—was effected by a number of men, twelve or fifteen perhaps, a majority of whom were members of this body, and had received their training in it, in Association work. The American Dental Convention had quite a number of the members of this body in its organization. That organization did not live very long; it was a kind of fast and loose affair; but after all it served a purpose in its day. It was the antecedent of the American Dental Association, and closed its career within a few years after the organization of the latter body.

Both of these organizations received their impress, and their formation largely from what had been done in this body, as a pioneer in this work. Now, what has been done by some of the men who were in it? We all use the mallet, for example: The first conception of it, the first mallet that was produced, or, perhaps the first two, were by members of this body.

The late Dr. George W. Baxter, of Warsaw, Ky., devised and constructed, so far as I know, the first automatic mallet that was ever made. Contemporaneously with him, and I think independently of him, there was an automatic mallet constructed by Dr. Collins, who resided in this state, both these gentlemen being members of this body, and were such for some time previous to that. The man who introduced the mallet to the profession, as an instrument in filling teeth, was a member of this body—Dr. W. H. Atkinson—introducing that implement first at a meeting of the Indiana State Dental Society, so far as I remember, in February, 1859, or 1860, was it? (Dr. Wright: I think so.) Perhaps in February, 1859; I am not sure, but it was one of those years. He was a member of this body, who did good work for it while he retained his membership; he was a member for three or four years. Some of the best work of his life, so far as I have ever heard, was done in this body. He was very advanced in his thinking, and in his work, and his influence made an impression on the Society that has never wholly passed away. This Society was the first one to offer a prize for a popular essay on dental surgery, looking in those days to giving intelligence to the people as a means of elevating the profession. The men composing this Society at that day recognized the necessity for disseminating such knowledge in thus offering a prize of one hundred dollars for the best essay—popular essay—on dental surgery, with a view of educating the people, with a view of making their influence felt abroad among the people, believing that the result would be the elevation of the profession, and the broadening of the popular appreciation of the profession, and the benefits conferred by it upon mankind.

This Society in those days proved itself able to do what no other Society could do. This Society, for example, took up the full consideration of the amalgam question and also that of dental patents. It took advanced ground on those subjects, having them under consideration for three years or more. They discussed these subjects thoroughly, but after all the Society was not injured; it was really benefitted by this discussion; but what was the result in other societies of the presentation and discussion of this same subject? It was upon the discussion of the amalgam question that the American Society of Dental Surgeons, one of the first organizations in this country, foundered, and went to pieces—on the dissensions and troubles that arose from the discussion by them of that question. The same subject was discussed in this body calmly, dispassionately, intelligently, thoroughly, and notwithstanding there was a diversity of opinion, yet the discussion was carried on so thoroughly, so fully, yet without injury to the body. It was, as I have stated, a positive benefit to it. It demonstrated its inherent strength that it was able to survive other bodies seemingly much stronger, but which had gone down nevertheless, simply because of internal dissensions arising in this way.

These are a few of the things that have been done by this body. A number of teachers have gone out through the influence of this body, who have been active in educational work for many years. In most of the more important societies organized years ago the members of this society took more or less part, and made more or less of an impression; they were able, by their experience gained here, to exercise an influence for the benefit of other societies. In the early days, many a time use was made of the constitution of this society for the information of other societies in many points. The experience of this society was a guide to others in very many particulars; at least it was in the early days sought for quite extensively by others who were taking up Association work.

These are only a few of the things that come to my mind in thinking over the work of this Association; but is not this a grand record? Is it not the proper thing to sustain this body, having a record of this kind, and not to simply lay it upon the shelf, and say: "You have finished your work; there is no longer anything for you to do." It seems to me that this Society in remembrance of its past history, of what it has done, that it ought to buckle on its armor and go forward, and stand in the front ranks to-day, and in the years to come, as well as it has in the years gone by. Stand in the front rank! Be a leader, as it has been the pioneer. Let it rather continue to be the pioneer for future organizations, as well as for those of the past, in all that can elevate our profession and advance its highest interests. Shall it not be sustained? Shall it not go on with the grand work that has been so nobly commenced and so persistently carried forward during the last half century?

I leave you, gentlemen, to answer this question.

Dr. Wright: Dr. Taft has well spoken of this Association as being the mother society. It is also the mother of one of the most active and useful societies in Europe; the American Dental Society of Europe can claim the Mississippi Valley Association as her mother. Over twenty years ago five men met on the Righi and acted as midwives for a little boy that was born to this society. All of those gentlemen were members of and received their training in this society. They were Drs. J. G. Van Marter, George W. Field, C. T. Terry, N. W. Williams, and myself. And this little society, conceived at that time, or born at that time, on the Righi, was baptized in good champaign, and has been a very thriving and useful society ever since, and it would be very sorry to hear of anything amiss happening to its mother out here in Cincinnati.

DR. HARLAN: As I am one of the younger members of this society I cannot indulge in any reminiscences to any large extent; but I would like to say that among the names of those embraced in the membership of this society I now recall, who have not yet been mentioned, Dr. S. P. Cutler, Isaiah Forbes, Eward H. Hale, Henry Barron, C. W. Spalding, W. H. Eames, and others in that locality, who have all deceased. Isaac Knapp, the leader in the State of Indiana for many years; and one of the old familiar faces that I remember, that I see no longer, is J. P. Ulrey; and then in Ohio there was ——— Rankin, one of the staunch supporters, and George W. Kelly, for many years Treasurer of the American Dental Association; and I might go on and mention names of others—S. J. Cobb, of Nashville, and Ross, of Kentucky, and Wm. H. Goddard, of Louisville. If I were to continue I might mention many other names of those who were members of the Mississippi Valley Association of Dental Surgeons, men who contributed largely to making dentistry what it is to-day—a respectable profession among the other professions. Many of the famous dentists of Europe have been members of this society, men who have practiced there in times past, and retired, and some who have gone hence. I remember Blount, who was a member, who now lives in this country; and Jenkins, of Dresden; and Hedden, (?) who formerly lived at Madison, Indiana, and still others; one man from Ohio, Dowd, lived in Paris for many years. I am not quite certain if Dr. Bing was ever a member of this Association, but Shelley was, and Blandy, who still lives in London; and the elder Coffin, who was a member of this Association for a short time, but is dead, and leaves his sons in the practice in the city of London; and then over in China there was H. H. Winn, and other men who lived in

China and Japan; and all along there through the islands there are men who were members of this society. One of the very young men, a son of the well-known dentist, is now practicing in Shanghai, J. W. Hall; and W. St. George Elliott, who practices in London; and in Tokio you will find a member of this society who was a frequent visitor at its sessions in years gone by; and in Rio Janeiro there are one or two men who were formerly members of this society. I might go on; but I will not. J. M. Whitney, of Honolulu, was a member of this society many years ago. I think that is a record of which we should be proud. Some of the very best literature that the dental profession possesses has been contributed by members of this society; and if they do not always give their productions to this society in the first instance they read them before State Associations throughout the United States, and they read them before the National organizations; and it may be safely said that it was the stimulus derived from their connection with this society, to which they largely owe the inception of these literary labors. And it was largely through the inspiration of this society that we owe the present state of dental legislation throughout this country. Ohio did not have a law, I believe, in regard to the practice of dentistry, until 1867 or 1868, somewhere along there. If she was not the first State, she was one of the first, to adopt such a law; and since 1867 every State and every Territory in the United States has a law regulating the practice of dentistry; and since that period every foreign country has taken the cue from us and has adopted rules and regulations covering the practice of dentistry. It is not possibe now to practice anywhere in Europe, except in Asiatic Turkey, without first complying with the local police regulations. England is far behind us; she did not enact a law until 1878, and it is only within three or four years that France has had a law regulating the practice of dentistry. Switzerland, two or three years prior to that; and Italy one in 1890. So all these things have come practically from the United States; and it does not matter if once in a while the biologists and bacteriologists of the United States have not been in the advance guard.

President Taft: (Resuming the Chair.) Has any other member anything further? Dr. Betty, shall we hear from you?

Dr. Betty: I think I said enough yesterday on that subject,

President Taft: I received a telegram from the President of the American Dental Association, in which he sends greetings to this body, and remarks that he expected to be here until the last moment; hence sends greetings, and asks that this body appoint delegates to the American Dental Association. Drs. Welch and Emminger are the members of this committee, and I will ask them to select the delegates from this body to the American Dental Association. We are entitled to one for every five members. I would suggest that those who think of going will report themselves to this committee, to save the committee the trouble of hunting them up, and that they so report as promptly as possible, that they may be properly appointed and duly authorized to go as delegates from this body. The national organization meets at Asbury Park on the first Tuesday in August; there will be a grand meeting, and no doubt a very important one, one that everybody is always interested in when they are there, and you should all be interested enough to attend. I have a letter from Dr. W. M. Morrison, in which he says: "My compliments and congratulations to the Society, and sorry I cannot be with you on the occasion of this meeting. Sincerely hoping it will be a successful and profitable one, I am, yours truly, Wm. M. Morrison, of St. Louis." He is one of the oldest of our members, if not the oldest; and it is something of importance that is keeping him away. I just received a letter from Dr. W. P. Hall, one of the old members of this body, a portion of which letter I will read: It is dated from Piqua, Ohio.

"I deeply regret that my engagements are such that I can not be with you at this meeting of the Mississippi Valley Association. I think I became a member of it in 1855 or 1856, and maintained my membership until within a few years, (three or four years; Dr. Hall was here, I think, three years ago, perhaps.) The annual meeting and greetings and discussions and clinics were to me always pleasant and profitable. I consider the Association as a great educator, and very helpful to all live progressive men in the profession. Fifty years ago the first day of this month I commenced the study of dentistry with Dr. John Jones, of Dayton, Ohio, who was quite distinguished as a skillful dentist in his day. His early practice antedated metal dies and swedge plates, even before porcelain teeth were made, except pivot teeth. I remember seeing a partial upper set of teeth he made for a lady, by bending gold wire and adjusting it with pliers to the mouth and remaining teeth, and next forming clasps around the molars, which had been worn satisfactorily for twenty-five years. The teeth were soldered to the wire; I presume you have seen many such cases. For several years after I commenced practice, we used spiral springs to hold the sets in the mouth. (Perhaps many here do not remember of having seen work of this kind.) I spent two years with Dr. Jones, and on the first day of May, 1847, I located in Piqua, where I am still happy in the daily practice of my profession. This fiftieth anniversary naturally recalls the past, and in the retrospect we may well be proud of the progress made in dental science in fifty years. Indeed it is almost phenomenal. Wonderful improvements have been introduced, and yet some of the old practice will bear favorable comparison with the new. You will pordon me for mentioning one or two illustrations, personal to myself. A short time since, a lady from Urbana was visiting friends in Piqua, and she called to see me and show me the teeth that I had made for her so many years ago, and they were still doing as good service as ever. The upper set I made on gold forty-three years ago, and the lower set is continuous gum, made thirty-three years ago. Neither require repairs. How many of the old members can show a better record? I may possibly get down on Thursday. I hope you will have a delightful session. Salute the brethren, and believe me ever, yours truly."

Dr. Callahan: I have received a number of letters of regret from those not attending. Some I have not yet opened. Among others I find one dated April 17th at Indianapolis, in which the Executive Committee of the Tri-State Dental Meeting cordially invites members to meet at Detroit, Michigan, June 18th to 20th next. Signed by G. E. Hunt, Secretary.

Dr. Welch: I have the name of Sarah S. Harris, graduate of the Ohio Dental College, a candidate for membership.

On motion, the rules were suspended, and the Secretary was instructed to cast the ballot of the Association for admission to membership of the lady named. It was so done, and announcement formally made to the society of the fact.

President Taft: Dr. Harlan has placed here a couple of volumes, embracing the transactions of the Columbia Dental Congress, a very valuable work, indeed. He states that there is a number of sets on hand still, and that can be procured if any member wishes the opportunity. I can say this: I received the work a little while ago, and it is a most excellent work, one indeed that no progressive dentist can afford, in justice to himself, to do without. I contains a large amount of very important matter indeed, matter that will serve for reference, and good reference, for years to come,—all the lifetime of any of those present. This work is still procurable. There are about three hundred sets remaining, and they can be had at the price of membership in the Congress—ten dollars; that is in leather binding; the price of which is twelve dollars, a mere pittance for the value that will be obtained.

Dr. Smith: To those who attended that meeting it will be almost essential. I want to say that Dr. Harlan, who edited that work, has done it admirably well; and he has done it without compensation. They are short of money about $1,000, and anybody who feels like contributing in purchasing these volumes will be doing a good thing. The American Dental Association at the last meeting made an appropriation of $500 to help pay for this work; it has been expensive because of the large number of illustrations. Take such a subject as was treated by Dr. Callahan yesterday; it gives you an extended paper on the subject of tube teeth. So it is practical, as well as embodying professional research.

President Taft: I am sure any subscriber will have more than the worth of his money. It will not be a contribution.

You will receive more than the worth of your money. There should be a vote of thanks extended to Dr. Harlan.

On motion it was so ordered.

Dr. Brown, of Ft. Wayne, Indiana: You are, most of you, aware of the effort now making to erect a permanent memorial to Dr. Wells, who discovered nitrous oxide gas as an anæsthetic. That those not informed may understand, I will read the following from a circular, which is issued by the committee appointed by the American Dental Association:

"At the celebration of the 50th anniversary of the discovery of anæsthesia, held at Philadelphia, December 11th, 1894, a resolution was passed directing the President of the American Dental Association to appoint a committee to take into consideration the erection of a suitable permanent memorial to Dr. Horace Wells, and to secure contributions toward the fund for that purpose. Dr. J. D. Thomas, of Philadelphia was elected Treasurer of the meeting. It is hoped to secure enough money to erect a bronze statue of Dr. Horace Wells in the National Capitol. Details as to style and character of the statue, as well as its definite location will be decided on at the next meeting of the American Dental Association to be held at Asbury Park, New Jersey. You are invited to contribute whatever amount of money you may feel willing and able, to the fund, etc. It is hoped that a motion to vote a suitable sum from our Treasury in behalf of this worthy object will prevail. Our Association cannot well afford to withhold its name and influence, as a pioneer organization, from this cause."

President Taft: You have heard the statement by Dr. Brown. What is your pleasure in regard to it?

Dr. Smith: Would it be proper for this society to make a donation?

On motion, it was resolved to contribute ten dollars to the fund for the erection of a permanent memorial to Dr. Horace Wells; or more, if the state of the Treasury, in the opinion of the Executive Committee, will warrant it.

In putting the question on the foregoing, President Taft said that he hoped that this formal action by the society would not preclude other contributions by members, but that all would contribute according to their abilities: If they could not spare a dollar or five dollars, then give less, as the cause was a worthy one.

PRESIDENT: The next order of business is number 5, on our program, "Crown Work," by Dr. George Evans, of New York City.

DR. EVANS spoke as follows:

Mr. President, and members of the Association, I would say in explanation of my not appearing before you with a regularly prepared paper, that I was notified by your committee to give a talk, or as I understood it, a demonstration, on the subject of something relating to crowns or bridge-work probably that would interest you a little; and it was not until I received the announcement of the meeting of the Society that I noticed my name was down as an essayist. I have not had an opportunity to prepare a paper, as I stated, in regular form, and will have to depend upon notes in the rough, etc., to guide me during my talk, which will be a criticism of some forms of crowns for front teeth and an explanation of a method of crowning. I will preface my demonstration proper with a few remarks, which to some, may appear irrelevant, but are not so, I think.

In presenting the subject of a tooth crown, I am aware that dental science, in the opinion of many members of a dental society should receive preference to that of art. But the usefulness of the profession depends on its art. Science is its guide, its aid. This statement, however, is not disqualifying to dentistry, respecting its acceptance as a profession. I so remark in consequence of some opinions that I have heard expressed here upon yesterday at the session of this society. What is science, and what is art? I will endeavor briefly to explain. Nature is the presentation of the phenomena of the universe, including man himself. Science embraces the study, the investigation of these phenomena; art is the utilization of these phenomena of nature, and the reproduction of them. Science, to further its investigation, depends on art; art, on science to still broaden its field of usefulness. For advancement one is often entirely dependent on the other; for, in science we use art, and art in science. Art embraces everything that is directly or indirectly the result of the work of the hands, including the creations of the sculptor, the painter, the architect, the physician and surgeon, the dental surgeon, and so on down to the humblest mechanic. The medical profession is constantly practicing art. What is a surgeon doing in the formation of a splint or appliance for his patient if it is not art? Or in the construction of a plaster or wood fiber jacket, in cases of spinal curvature, on his plaster model of his patient is he not practicing art?—indeed that which may be decidedly pronounced mechanical, so far as art is concerned. Dental art consequently, should receive the same liberal consideration in the classification of dentistry under the head of a profession, or in placing it as a specialty of medicine. Therefore, anything that is at all novel in art, or that may be of interest in that connection is proper for presentation to a dental society. Now, I have here drawn the form of a front tooth, a central incisor; the presentation of that to you, the mere drawing, is art; but the consideration of that form, analytically, is science. Now, that is simply a drawing of a central incisor; but there are some points about that even, that some of the gentlemen here have not considered. In that drawing we notice the apex of the root and the incisive edge of the tooth are almost on a direct line. Now, there seems to be nothing much, at a glance, to assist us; but there is a great deal to guide us; in the first place every tooth crown that is inserted should have the incisive edge, as nature intended, directly on a line with the center of the root canal, the root on a line with the apex. Place it the least bit forward or inward and you throw it in an abnormal position. That is a guide to us in drilling the root, not only in a front or incisor tooth; but it is also a guide to us in reaming the root canal; if we follow that guide it will prevent us from perforating the side of the root. All you have to do is to calculate where the incisive edge of that tooth was originally placed; also guided by the eye as to the position of the adjoining or approximal teeth's incisive edges, which will control the direction of your drill or reamer. I mention that in connection with the method here illustrated regarding the crowning of a front tooth. The objectionable features of collar crowns are the exposure of the collar at the servico-labial section, which is difficult to avoid, and the irritation its presence there is apt to cause the peridental membranes, which it is more liable to do at this point than at the other sides of the root. The collar, to be invisible, has to be fitted well under the gum-margin. This requires extensive removal of the periphery of the cervico-labial section of the end of the root rendering adaptation of the collar at this point an operation few practitioners succeed in accomplishing perfectly. The width of this section of the collar has also to be so reduced that it contributes but little strength to the crown. As a matter of course, in bridge-work it is almost absolutely necessary in many cases that we should have the collar to entirely encircle the root of even a front tooth, but I tell you, many times patients will not endure the exhibition of the gold outside the gum margin. It is often almost impossible to avoid this, in many cases, if it is placed there at all. Many of these patients are ladies who have visited Europe, and have gotten European ideas instilled into them in regard to metal showing, and they must have a plate put in having a crown that the gold shall not show above it. This is not so all over the country, but in many sections it is the case, at least with the society ladies of New York city. And I don't think it is confined to New York city. Another thing, we will consider, is, what is the use of a collar on that portion of an upper incisor tooth, and I refer in my discussion here to-day almost entirely to the upper front teeth—I am not attempting to bring before you an exhibition of the crowning method in all its details. I wish only now to bring to your attention a few points, and I must necessarily be brief. We all know that in the normal occlusion of the front teeth that the force comes as exhibited below. (Referring to illustrations on paper in view of audience.) I may say that this method of presenting these drawings on a roll, like this, is a method perhaps a little different from what you have seen; but I find it very convenient in lecturing to college students, because it gives me a cue to my subject, as I go along, and you can easily withdraw from the sight of students the portions discussed and have only before the eye what is being especially considered. That, I think, is a very good feature of it. It is also convenient to carry a large number of illustrations in this rolled form. Now, as I stated, the force of occlusion comes about in that manner—that angle. It will readily be understood that there is no necessity really at this line here for much support, beyond what is obtained by the cap itself. All the resistance is required at the palatal section of the root. The form of crown that I here illustrate is not new in principle, neither do I present it as a universal substitute for the ordinary collar crown, but as embodying features which are advantageous. The method I offer facilitates and simplifies the operation of construction so that it can be performed by those of only ordinary skill. In my intercourse with dentists I have had several very prominent men in the profession candidly, privately acknowledge to me that they never could quite, satisfactorily to themselves, fit a collar on a front incisor tooth; in fact, on many teeth. There was always something about it not quite satisfactory when the operation was completed. Now, this embodies the principle of the collar crown; a collar is not necessary, if you can have a perfectly tight, accurately fitting post in the root, and in the metal or cap which covers the end of the root, that is all you need. You can obtain all the strength necessary in this manner. Dr. Van Wort of Brooklyn, seldom, as I understand, places a collar on the root of a front tooth; I have seen some very fine operations, and apparently durable ones come from his hands.

One trouble with the collar as ordinarily made is this: there is a section right here at the cervico-labial portion of the tooth that is very difficult for many to fit their collar accurately to. In trimming off that edge, as is illustrated in the form of the tooth there, there is a curve that requires considerable trimming and reduction to enable you to properly fit the collar, and when it is fitted, there is many times an edge here that protrudes, as shown at "D," and when the crown and the porcelain then is fitted to endeavor to bring it in line with the other tooth, as in the drawing here of the natural tooth is illustrated, you have to protrude that porcelain a little further; that forms a cavity, or recess, that is shown here at "C," that in many cases is not filled in with metal. Unless you, in considering the curve, solder around the cap and collar in such a way that it shall flow and fill in up there you have got a recess that is more or less a detriment, and that produces irritation of the cervical margin, and causes irritation from that cause alone. In more than one case where recession of the gum has taken place, which I have examined, it was due to that little recess in the porcelain where it protrudes; it is not due to the collar. That is caused in fitting the collar, the tooth is removed at that point to get a perfect fit of the collar, and the porcelain is not able to fill it in; I don't say it is not possible, but it is difficult, unless you run your solder in there it is always difficult; you will have that little recess that will constantly produce the irritation. Now, take a superior incisor, as here illustrated. This shows the form to which the root is to be trimmed. The end is trimmed at the proximal sides and sufficiently here at the palatal side to bring it on a line with the gum; the cervico-labial portion is left intact. In doing that I use these trimmers, such as I will here show you; they have not before, perhaps been brought to your attention. They have been introduced by myself; I have tried every other form that has been presented by others, and after all I have never found anything—and other gentlemen have endorsed that opinion to me—anything equal or superior to these for trimming on the sides of the root, placing them in a dental engine, and resting the finger on the incisive edges of the adjoining tooth you can trim off the root most easily. I also use, in trimming the sides, and instrument like this, which aids me very much. (You will excuse me in introducing these instruments in the way I do. There are a good many questions asked me by gentlemen, how I do this, and how I do that.) After the shaping is done in the manner indicated, with disks and trimmers, the root-cannal is next enlarged with an Ottolengui root-cannal reamer, which only takes a moment. The size and condition of the root and the judgment of the operator should determine the number or size of the reamer to be used, and the depth to which the canal should be reamed. I generally commence with No. 1, the smaller size, and then increase. I very seldom use one larger than No. 1 for an incisor, and No. 2 or 3 for a cuspid. To the reamed canal is fitted a prepared iridio-platinum post, as illustrated here in Fig. 6, corresponding in size to the reamer used. By reaming the tooth-canal and then introducing the post, if it is exactly the size, you at once have a tight fitting post. By reason of the serrations or screw thread on the post, if you draw out the reamer it will enable the post to permanently and rigidley fix itself in the canal. That is one point gained; and the object of this is so that it can be screwed to this platinum plate. Next take this prepared platinum plate, having a perforated concave depression. Its size to be proportional to the root of the tooth. The depression is formed in it as illustrated here. It can be stamped on a die; one form will almost do for all cases, because the plates, if made a little large, can be trimmed down on the edges to suit. This depression I fill with a small piece of pure gold, then punch a hole in it, and enlarge it so this screw post will fit in it. Some of these disks and posts are here presented; the gentlemen can pass them around—the reamers at the same time. I show them because some of the gentlemen never have used them, and it is necessary to call attention to them. If necessary the orifices may be slightly enlarged with a round headed bur. The disk of platinum plate is about No. 35 gauge. In the depression of the disk the pure gold has been mounted, and the post, when fitted to the canal, is grasped at the line of the orifice of the canal with small pliers, about here, as you see, in that manner (illustrating). That gives about the distance it goes into the post. The disk should fit in the orifice of the canal, when the post is in position by twisting, the post is then put into the plate and the edges turned down. By twisting the post in the disk, change of position is instantly effected; some times, when just about nearly through I have found a chance to give it a press upwards, which will bring the edges of the platinum down into the orifice of the canal, and the serrations on the post enables the post to catch firmly. Both post and disk are next removed, and the post secured in position in the disk by being held in a Bunsen flame, and heated to a point that fuses the pure gold in the depression.

No flux is necessary as sufficient remains from the first fusing of the gold. The post with the disk is next inserted on the root, the platinum pressed with a large flat plugger, and malleted so that the line of the edge of the end of the root will be impressed upon it. The platinum is next removed, and slit at the two points between the palatal and proximal sides. The line of the end of the root is supposed to be represented here on the disk. By the process I have just explained, and guided by the mark of the end of the root on the platinum the proximal portions are bent over with small-pointed pliers to embrace the sides of the root. The post and cap are then placed on the root and the side flaps, with the aid of foot-shaped condensers and burnishers, are closely fitted. The palatal flap is next brought down to position. Frequent removals and annealings are necessary during the process, which should include finally trimming the edge of the platinum, smoothing with a corundum point, and then an annealing and an all-around burnishing of the cap to the root. At the cervico-labial section the porcelain can rest on the platinum, or the platinum can be trimmed so that the front edge of the porcelain may be fitted against the root, covering it, the platinum is trimmed off at that point. The projecting end of the post should also be removed, leaving it a little flush at the palatal side. The porcelain front, which should be a cross-pin plate tooth, is ground and closely fitted to the surface of the root or metal, as may be, at the cervico-labial section under the edge of the gum, but a properly shaped space opening toward the palatal side is left between it and the surface of the cap. To shape the porcelain simplifies the fitting of the cervical section. The space between the cap and the porcelain is also easier filled in the soldering. To the porcelain front, here, a piece of very thin platinum foil is shaped, the porcelain heated, the part veneered with a mere film of gum shellac, and by pressure with a napkin or cotton the platinum foil is attached thereto. The rest of the porcelain is then backed with the platinum plate (about 35 gauge). The platinum is left slightly extending over the incisor edge, and the porcelain front is waxed in position on the cap. Fig. 13 shows the crown waxed up ready for investment. Wax in full amount must be extended over the collar to its edge, in the seams, and between the porcelain and the cap at every point solder is to flow. I generally use Parr's wax. In trimming the investment the material must not be removed from over the collar lower than the line of the surface of the cap, or in such a manner that the platinum turn-over edges are not exposed to the direct force of the flame. If the collar is not exposed, the solder will flow over the outer surface of the platinum, just where it is wanted and where wax has been applied, and all the parts will become united.

The investment must be slightly raised at one end, and headed up at its base with a full flame of a gas blow-pipe thrown in the direction indicated in the drawing. Heat thus applied will cause the solder to flow downward and fill the interstices in all parts of the envestment as though it were an ingot. The best way is to apply a little solder at a time until the deep parts are filled. The flame is then withdrawn for an instant, and with a small pointed flame and more solder the backing can be contoured. As platinum forms the cap and backing, the soldering can be conducted without fear of accidentally fusing those parts.

I wish to explain a point with regard to the cementing of crowns, this kind of crown, or any kind of collar crown. Having the root and crown ready I warm the crown, and apply a thin coating of chlor-gutta-percha to the post. The chloroform, instantly evaporating, leaves a film of heated gutta-percha. Immediately the crown is adjusted to the root and removed. This shapes the gutta-percha on the post. The crown is then allowed to cool, and is cemented on as though no gutta-percha was used on post. A crown so cemented can be removed at any time by repeated applications of the thick part of the heated root-canal drier to the metallic portion of the crown, which communicates the heat to the post. In a short time the sheath of gutta-percha around the post is softened, and the crown can usually be removed without difficulty. I also attach ordinary bridge-work in this way, having abandoned the use of methods classed as "detachable," which only allow the bridge to be removed by the dentist. (Dr. Evans here passed among the audience a tooth which he had crowned and afterwards had to extract, which exhibited a sample of his work that was made without any idea that it would ever be exhibited as such.)

The question may be asked, as it was asked of me once by a gentleman, when I was describing this method, does that pin enveloped with a film of gutta-percha hold in, in that way? You all know you can cement on a crown very firm, if you have a nice fitting post, with a little film of gutta-percha alone. Here is an ordinary root that has been capped in the usual way, and the post alone is secured with gutta-percha, and nothing put in the cap; try that and see if either of you, gentlemen, can move it. I question if I could move it with the pliers without heating it. In that way I have found perfect satisfaction and security.

One other thing I would like to bring to your attention, which I have had remarkable success in, and that is in making all-gold dummies. A great many gentlemen have told me that they preferred to use on the lower jaw where it is not seen, gold dummies. We all know that in making gold dummies with a porcelain front they fill them in at the labial side in the cap, with gold, that makes them very heavy. Where they make them entirely of gold, like the dummies of the kind shown to you in the past by Dr. Knapp, of New Orleans, you remember they are heavy. My method is to form these dummies hollow. You can take a crown—I prefer a seamless crown that is stamped out of one piece of gold. You can do it with one of those crowns you can purchase, you can do it with a crown that is made with one of the machines now in use. The crown is shaped and fitted to the model as a dummy, and, if you intend to have it rest on the membranes, in the form that the bridge was constructed yesterday that was shown by Dr. Callahan; you can then remove it and place it—first, before that, though, I fill in the crowning surface with gold solder, or gold plate—the crowns that I use are alloyed with platinum—you can melt gold plate right into it and fill the space in. I always boil them out in acid. I never solder the crown on the outside, till I remove the flux; that is a little point. I don't want to melt them; it spoils any bridgework. Don't be in such a hurry that you neglect to boil them in acid, and remove that flux from the inside. If you neglect that you are very apt to melt them when you solder on the outside. I remove that and then adjust this ball of solder in the center of the flame and then heat it up to the melting point, the solder flows down and runs along the plate, and the moment it strikes the edges it firmly unites. The point has been brought up. "Is that hermetically sealed up? Does it leak?" To an educated man I should think it is not necessary for me to say it is hermetically sealed, because we all know when you heat that to a certain point, to a red heat, you will exhaust almost entirely the air. When you heat to a dull red heat your solder melts, then you heat it up a little further and the solder flows over to those edges, and as the crown cools, as a matter of course the solder cools, and the entrance is instantly stopped, and hermetically sealed—just as a housewife, as you all know, takes a jar of preserves and fills it up with boiling water and seals down the top. Make the form in this way. This is to make a certain rim that I have not heard much said about, but which I see in many men's work, and I practice in my own work. It is all right to restore the teeth in form, but I prefer to favor the grinding or occluding surface of a dummy always; consequently from the palatal to the labial side I always reduce the dimensions of it slightly, about in proportion as you see there; that is the normal average size of the tooth, (illustrating) and that would be the proportional size, as a rule, I would form my dummy.

There is something else in that. It lessens the curve of the self-cleaning surface. It is very desirable not to have too much curving in. If that was a long tooth it is very apparent that an immense slot would be formed by making the dummy as wide in proportion as that. I will pass around some specimens I have here. There is a bridge I formed when I was getting ready to go to the American Dental Association last summer. I was not over a couple of hours making that bridge. As a matter of course I could not do that, with work intended for the mouth.

In making my explanation you will pardon me if I have presented any material such as I have presented at the American Dental Association's meeting. I have not before presented it as much in detail as I have here. I presented it there and afterwards made some little drawings. My explanation there was anything but clearly understood by the gentlemen present, I fear. It has been commented on favorably by my friends, and Dr. Crouse thought it would prove interesting matter to you. As I replied to Dr. Callahan, in asking me to appear before you, such an educated and intelligent body of gentlemen as the Mississippi Valley Association, it would be very difficult for me to present to you anything in the way of crown or bridge work that would be novel or interest you. I did not wish to encroach upon your time to no purpose, and have selected these few points, hoping you would find something in them of interest. Because of this method having been favorably commented upon, I have felt encouraged to present it. I thank you, gentlemen.

Dr. Taft: The remarks of Dr. Evans have been very full and exhaustive and I presume very satisfactory, yet I think we can well afford to devote a few moments to further discussion upon this subject, it being a very interesting one. Has any gentleman anything to offer?

Dr. Grant Molyneaux, of Cincinnati: It is certainly very gratifying to have members of the profession of the prominence of Dr. Evans come from New York city to Cincinnati and give their valuable time to us in such an interesting address. I have heard some of his remarks before, and have found many of them to be extremely valuable. The talk he gave us embraced several very important features, but there are some other features that I don't quite agree with the Doctor in—one point first, in connection with the crown, I would like to ask him whether this crown would be servicable in connection with the average, porcelain crown? Before he answers the question, I would say that we believe the object of a ferule among the profession, many believe as the Doctor has stated, that the ferule is necessary to make a strong crown—and I am one of those persons who hold that in the majority of instances a ferule is necessary, especially in bridge pieces; and I believe that a complete ferule is necessary, if we are to have a ferule at all. The object of the ferule is to support the arch, give the greatest strength to the arch; and at the same time that a half ferule does not fulfill the object of the ferule in all instances, and that in order to add sufficient strength to the crown a complete ferule would be necessary. It strikes me that in the majority of instances a ferule might completely encircle the end of that root without destroying any more of the root than the Doctor does in the adjusting of this porcelain facing. I do disapprove of attaching ferules to any extent beyond the free margin of the gum. Take a root of a tooth in that shape. Now, of course, if the root is left in that form, it is usually, in fact it is quite impossible for anybody to pass below the free margin of the gum with a ferule; but is it necessary, does it add any strength to the tube to pass far below the free margin of the gum? It strikes me it is only necessary to reach this point, just below the free margin of the gum, and at that point we can slightly cut those corners, and adjust a ferule to that point, and to that point, (illustrating) completely encircling the root. It would add quite as much strength, in fact, just as much strength as if that ferule passed below an eigth of an inch, for the strength given to the crown by the ferule is only at the point where the ferule is in contact with the arch, and beyond that it becomes a source of irritation, and a receptacle for stray food that might decompose and give rise to trouble, as frequently happens where this ferule is driven far below the gum. It seems to me, that in taking this ferule off here, as the Doctor suggests, we lose to some extent the utility of the ferule, that the root is not thoroughly protected, and that we will meet with difficulty from attempting to adjust a porcelain crown ground to fit close against the root, without any protection to the margin. There is another point that is in favor of the operation, and that is, its artistic feature. That the method Dr. Evans has suggested here is probably one of the most artistic crowns that can be adjusted. But would it be a serviceable crown, would it be a strong crown, in connection with bridge work? Of cource the object of of a ferule is to give additional support to the pin by impinging upon the sides, and only in that way, as pressure would be exerted at this angle (illustrating) of the tooth, from the lingual side towards the labial, that the ferule would bind and give resistance, and an additional support to the pin, by pulling against that angle, so it has been hoped, as this force is the greater in the direction I have indicated, and the ferule would compensate that. We extend our ferule that usually make it heavier, and as the gold is not to be seen the tube is allowed to project a little below the free margin of the gum, and formed in this manner, so that we can get a firm adjustment of the ferule, while anteriorly we reduce it to such dimensions as that it shall be hardly perceptible. That gives it a large degree of support. There might be some instances in connection with bridge-work, when the force would be exercised at this angle, and then this half ferule would fail, and this tooth fixed in that manner would not give support, because the angle of force, biting against that, it would push the crown in this manner, and the ferule would not give the support intended. That would be more complicated bridge-work. They would probably put a single crown where protected on both sides by the natural teeth; and where the force is distributed, or between several natural teeth with the crown adjusted just as ordinarily. Now, then, it seems to me that there is another point that is of value, in suggesting a full ferule for the teeth; and that is, to make this joint perfect. Take another root, and if we allow the ferule to pass entirely around the tooth, then grind in our porcelain facing, fit this and back it up with thin platina—that has been my hobby for a long time, to back up with—either thin platina or pure gold. There is a peculiar point—where the color of the teeth is a yellowish or cream tint, and you line the teeth up with platinum, porcelain teeth, well—at night—that tooth will be conspicuous, because it will look like a discolored pulpless tooth; it will be a very dark tooth; if it is lined up with pure gold it is not so perceptible; so that is a point to be considered. If a bluish tint is to be developed in the lining up of porcelain teeth with a platina back, it is suggested that if the teeth are cream color pure gold would be suggested. I had an experience of this sort that caused me a great deal of trouble. A lady complained, said her friends told her she looked like she had a dead tooth in her mouth. In day time it was not noticeable, but at night it was distressing to her friends. I tried it again, with the same result, on a lighter tooth, until I used the gold and then we seemed to get the color better proportioned. If we surround those teeth entirely by a ferule I believe we can make a more serviceable piece of work if we allow our ferule to come a little higher; that is, encircle the root entirely and then let this backing come over and barely lap the edge of this tooth at this point; then, before adjusting the tooth in connection with this band, painting that with a solution of borax, without allowing it to touch the porcelain, as our gold solder flows down from the plate, as he suggests, it flows on to this surface here, which need only barely face it and gives a very fine gold cap over that portion of the porcelain, which it seems to me is very advantageous in grinding. It requires some pretty accurate adjustment at times to do it, but in many instances it can be done, and then the joint here is perfect. The solder flowing over it supports that tooth at the top, and the porcelain holds it at the bottom. It seems to me that the tooth adjusted here in this manner to the porcelain bearing, connected there, if the strain be in this direction, it might have a tendency to force the porcelain off the pins.

The suggestion Dr. Evans made in regard to a guide in drilling the root is exceedingly valuable. I have used that ever since I heard him last summer at Old Point Comfort. I have followed that advice in several instances with crowns that I was compelled to remove, and I found it to be exceedingly valuable. I could take a crown off very easily that while in place seemed to be perfectly firm. The point he made with regard to counter-sinking for the pin is another valuable thing.

I enjoyed Dr. Evans' remarks very much, and I think I can thoroughly commend what he said here, to the careful consideration of the most of my friends. Last year, at Old Point Comfort, the remarks he made there were so valuable that I put them into use. That one point about the ferule, I still believe in connection with bridge-work especially, it would be desirable to surround the teeth, and it is not necessary to get the action of the ferule to pass far below the free margin of the gum; therefore I would ask the Doctor whether he can depend upon that ground for the bridge as he makes it?

Dr. Smith: There is some difference in opinion in reference to the value of this ferule; from Dr. Molyneaux's standpoint, it seems it is not worth much. He says that the pressure is outward, and it is on the incisor teeth; when you have the band anteriorly the pressure outward would force the band away, would it not? It would not support it anteriorly, while on the root labial surface; on the contrary, if the impingement were outward it would bring it posterior, actually in contact, as it is where the support is.

Dr. Molyneaux: That is exactly what I said. I say in the majority of instances the force is outward, and therefore it would not be necessary in a single crown to have any at all, but in bridge-work, if the force is directed from outward in, is protruding much, then you would want the ferule on the inside.

Dr. Smith: There is no dispute about that. But it is the anterior tooth we are talking about; therefore it would indicate it did not need any anteriorly; therefore, if fitted accurately you have accomplished all possible.

Dr. Molyneaux: In bridge-work—where there is a lateral motion—take a cuspid tooth.

Dr. Smith: That is removed from sight much more largely than anteriorly. I want to call attention to the fact that the argument defeats what you claim; you take and support that pin by this firm attachment behind and then the crown he describes is certainly the one to make.

Dr. Evans: I will state that the crown is not presented as the suporter of bridge-work, although I have used it satisfactorily. You take a large, strong central, used to carry a lateral with it why, you will find enough support in this.

Dr. Molyneaux: You would not advocate it, then, as a rule, for bridge-work?

Dr. Cravens: We do not pay much attention to prosthetic dentistry. I suppose this comes under that head. Mark Twain, in his "Innocents Abroad," on the occasion where he visits Rome, had occasion to make the observation that it is astonishing how much you know under certain circumstances. He says, when he went to Rome and saw the Coliseum, "Now, I recognized it at once!" This crown, that Dr. Evans has given us a description of so admirably, I recognize as an old acquaintance. There are about half a dozen gentlemen, graduates of the Indiana Dental College, who, some of them, at least remember that crown as described, is practically the Cousins' crown. Some of them went so far as to call it the "Cravens" crown, which I disclaimed. I call it the "Cousins" crown. It is practically the same, the only exception being that the band is even less than given in the Fig. marked No. 9. In the crown I described and instructed students to make, there was just a little skim at the palatal or labial part of the root, the root being beveled directly from the front, in the upper teeth beveled downward, and absolutely no gold showing in front, in fact none there, even the platinum cap dressed back so far from the front that the porcelain of the tooth rested directly on the root. My objection to putting a band along the lateral surface, proximal surface, is that with those teeth there is almost always a depression laterally, and that it is the most difficult place to get a band to fit accurately. We can all fit closely enough in front, but with the proximal surface we have trouble; or in fact with any operation to be performed on that surface. So that I omit the band entirely, except just a little band. When Dr. Smith started out I thought he was going to make all of my speech. In fact, he did, except in regard to Mark Twain. I did not say anything about my Professors, but I want to say that I have made crowns that way as far back as 1880; but I don't consider it original with myself. I don't know who gave it to me first, don't know as anybody told me. That is, just enough over the palatal portions, in the direction as Dr. Smith referred to, from the rear upward and forward, and the little flange affords all the support you can possibly get. The credit of that crown was due originally to Dr. Darby, of Philadelphia, who twenty years ago gave a description of four incisors, which I think he describes as movable pivots, constructing four sockets for each, setting the sockets on the roots, pivotted and fitting tight. That was before the days of bridges. He describes that manner of making the crown. Dr. Marshall Webb gave a method of making the same crown, not removable, however, but fixed; and with Dr. Marshall Webb's admirable precision, he gives a method of making the crown so that when it was put on, it was impossible to put the crown in position with cement or India-rubber, or anything else, except in the position in which it had to go. I will add, that I object to the band going under the gum. In trimming the root—I construct it so that enough of the root, the posterior labial portion, should extend over the gum below, far enough so that the little section of the flange would not impinge upon the pericementum.

Dr. G. W. Smith, of Cincinnati: I just want to add a thought or two in the line of bridge-work. I see in this the same principle, I have in my office a crown I made ten years ago on the same principle. There is the same objections to this crown. It cannot be set up rightly for less than $25 to begin with; there was too much work about it. Again, in that crown, the main support, where it gave me the most trouble was, fastening the gold rightly to the porcelain. We are having that trouble to-day. The porcelain facing, without we can get up something which will give us more stability in the facing we have put on the gold, it will be carried away by mastication. That has been my trouble. I have been working on this for ten years. The best I can get out of bridge-work to-day is making the bands and suspension here described, and then melting the solid gold on them. Gentlemen, it is the acme process of bridge-work. It is the only thing that will stand; and I believe I may say that I make as much bridge-work as any of my dental friends in this city. I have found that in mastication it breaks down; then we have trouble to remove and repair it. It breaks down repeatedly. The only solid work is to do as has been done, fitting on those bands and then hanging solid 20 carat gold crowns between for the masticating back teeth.

Dr. Molyneaux: You can always find people who have seen similar things; but this crown they have not seen; I defy anybody to show it to me in the literature. There are some that approximate it, but they are not the same. Dr. Evans deserves credit for the many valuable points he has explained. The only point in my mind is whether it could not be a little better made by a ferule passing entirely around the root, but very narrow in front, and cemented down. It could be made of porcelain, with a platinum ferule, and you could fuse that instead of the gold with the electric furnace, such as will be exhibited by Dr. Custer. You will be able to fuse that whole surface with porcelain, and make a perfect operation. Dr. Evans has the credit for that crown as it stands; that is quite an advance.

Dr. Evans: The principle is as old as the hills; but the method, that is what I presented.

In answer to Dr. Molyneaux, I will say, that I have not presented this form of crowns as a crown for bridge-work. I think I have used it in conjunction with other support. I will say, that with a ferule surrounding three-fourths of a root, counting the lateral sides and the palatal side, all the force, in my opinion, and in that of most gentlemen, I think, excepting what Dr. Molyneaux has stated, is almost entirely in that direcaion. Now, as to hiding the collar, I can hide the collar, but, as I distinctly stated, it is a most difficult operation to perform; I have found decay occur where a root has been crowned without a collar. I have generally found it on the proximal sides, the sides that Dr. Cravens does not believe in crowning. In regard to the color, in lining with platinum, that is correct. I agree entirely with Dr. Molyneaux in that respect. Where you use the porcelain, as a matter of course, you can fasten those pins by first thinning it down and using the porcelain required in backing. In cases where this platinum lining has to be soldered a tint will be sent through the teeth by its use. Just before I left home the same question came up and I took a piece of platinum and rolled it out quite thin, and after shaping it as a backing annealed it several times to take the contraction in a measure out of it that takes place in the heating of these metals, and it worked satisfactorily. This is no new idea; I presented it in my work on crown and bridge-work. Even this very crown is there—just a little alteration in the method. About soldering over the edge of the porcelain here, that can be done, and I acknowledge I always endeavor to do it where I make the crown and put the collar there. My point in this was to make that little edge of porcelain take the place of the collar. As a matter of course you can trim that up and make it present a smooth surface to the investing membrane, or gingival margin. In regard to the proximal sides of the crown being trimmed down, I think the proximal sides very easy to trim down. I do it in an instant with a wheel. And now, in regard to Dr. Darby, and the question of originality, there has not been a crown introduced in the last twenty years that is realy new in principle. Oh, yes, go back over thirty—forty years—the old "Harris' Principles," that were put in my hands in 1859, has what you might call a gold pivot tooth; what is the crown of to-day but the same old thing, with a base of metal put around the line? I make such a crown as that, sometimes, on the cap end of the root, in this same old way, that was done fifty years, or a number of years ago, and after the crown is all finished, you might say—I then add a little base of metal around the palatal and part of the proximal sides. I invest it and solder it there, and it makes a mighty neat crown, too; but it is the same old thing, with a little collar added. As to Dr. Darby,—Dr. Darby was not anterior to these other methods; he is only one of the "Latter Day Saints on the subject of those crowns of his.

I have read of that matter, I think, very lately, though I have not especially looked it up; but I am well versed on this subject, and it is the same old thing, read of in the books away long ago. What is new in this is not the principle—the principle is old—away back—but it is only the simple method to facilitate the operation; and in speaking on that point I will answer the other gentleman, Dr. Smith, in suggesting that the whole point about that is to facilitate the operation. In regard to the time I will take, if you will give me the patient and the chair, and allow me, after preparing the root, I will ream the root canal, fit the post and adjust the cap in just ten minutes. I know I did crown a tooth before the Dental Society in about half an hour; I think those gentlemen, if there are any of them here that have seen me work, will believe what I say. I will let you take a watch, and I will make the die for the tooth in five minutes; and I am willing to show that process up too. I want to make this statement on time; I generally am on time.

Dr. Smith: Have you had any trouble with porcelain being broken away by mastication in the central incisors?

Dr. Evans: If you cover the incisive edge of your porcelain with metal as was described, the force comes on the edge. But this crown is not presented as a crown for bridge-work; but more as a method of crowning the largest of the incisors and laterals occasionally; and in ladies' teeth where you don't want the gold to show. Dr. Frank Abbott does not believe in collar crowning, and is not much of a believer in bridge-work; but he does believe in that crown. I think I advanced most of these points. I thank you, gentlemen, for your criticism. I like criticism; the more I am criticised the better I like it. A gentleman said to me once in New York, a microscopist, the worse criticism a man can get is no criticism at all; and that is just the way they treated me in Europe.

Dr. Smith: While we are on the subject of a collar: if there is any advantage in having gold contact, why not take No. 20 foil, gold foil, burnish down, and then place platinum on that, underlying your platinum back put on thick foil, and then form your back; then you will have gold, which is more desirable, I think, as some teeth are affected by metal contact.

Dr. Evans: That is an idea. I thank Dr. Smith for it; I will put it on record, and will practice it, instead of using crown metal; because crown metal is dangerous to use, the expansion and cooling is so unequal; in forming the collar you have often experienced difficulty in soldering the ends, and in lining the teeth with it, I have been afraid this expansion and contraction would crack and cause trouble.

Dr. Taft: Dr. Fletcher's paper is next in order. How long will it take, Doctor, for the reading of your paper?

Dr. Fletcher: I should prefer to have it read after dinner, and have paper and discussion come together. There is a paper here by Dr. Darling, that can be read; as I am one of the members to discuss it I can take up the discussion after dinner.

On motion the regular order was modified and the reading of the paper by Doctor Darling was made the next order of business. Dr. Darling being absent, the same was read by Dr. Callahan, during which Dr. J. Taft, temporarily resigned the chair to Dr. Emminger.

